# Antiandrogens as Therapies for COVID-19: A Systematic Review

**DOI:** 10.3390/cancers16020298

**Published:** 2024-01-10

**Authors:** Massimiliano Cani, Samantha Epistolio, Giulia Dazio, Mikol Modesti, Giuseppe Salfi, Martino Pedrani, Luca Isella, Silke Gillessen, Ursula Maria Vogl, Luigi Tortola, Giorgio Treglia, Consuelo Buttigliero, Milo Frattini, Ricardo Pereira Mestre

**Affiliations:** 1 Oncology Institute of Southern Switzerland (IOSI), Ente Ospedaliero Cantonale (EOC), 6500 Bellinzona, Switzerlandsilke.gillessensommer@eoc.ch (S.G.); ursula.vogl@eoc.ch (U.M.V.);; 2 Oncology Unit, Department of Oncology, University of Turin, S. Luigi Gonzaga Hospital, 10043 Orbassano, Italy; consuelo.buttigliero@unito.it; 3 Laboratory of Genetics and Molecular Pathology, Institute of Pathology, Ente Ospedaliero Cantonale (EOC), 6600 Locarno, Switzerlandmilo.frattini@eoc.ch (M.F.); 4 Department of Experimental and Clinical Medicine, University of Florence, Largo Brambilla 3, 50134 Florence, Italy; 5 Institute of Oncology Research (IOR), 6500 Bellinzona, Switzerland; 6 Department of Oncology and Hemato-Oncology, Università degli Studi di Milano, 20122 Milan, Italy; 7 Faculty of Biomedical Sciences, Università della Svizzera Italiana, 6900 Lugano, Switzerland; giorgio.treglia@eoc.ch; 8 Imaging Institute of Southern Switzerland, Ente Ospedaliero Cantonale (EOC), 6500 Bellinzona, Switzerland; 9 Faculty of Biology and Medicine, University of Lausanne, 1005 Lausanne, Switzerland; 10 Clinical Research Unit, myDoctorAngel, 6934 Bioggio, Switzerland

**Keywords:** COVID-19, SARS-CoV-2, antiandrogens, testosterone

## Abstract

**Simple Summary:**

The severe acute respiratory syndrome coronavirus 2 (SARS-CoV-2) pandemic recently represented an unexpected global issue. Due to its rapid spread and severity, the identification of possible therapies emerged as an urgent need. In this sense, the molecular landscape of SARS-CoV-2 infection was thoroughly analyzed to find possible therapeutic vulnerabilities. In such a context, the correlation between SARS-CoV-2 infection and antiandrogens was explored, finding promising but also contradictory results. In our work, we systematically reviewed the current literature to explore this issue.

**Abstract:**

Background: In 2019, the breakthrough of the coronavirus 2 disease (COVID-19) pandemic, caused by the severe acute respiratory syndrome coronavirus 2 (SARS-CoV-2), represented one of the major issues of our recent history. Different drugs have been tested to rapidly find effective anti-viral treatments and, among these, antiandrogens have been suggested to play a role in mediating SARS-CoV-2 infection. Considering the high heterogeneity of studies on this topic, we decided to review the current literature. Methods: We performed a systematic review according to PRISMA guidelines. A search strategy was conducted on PUBMED and Medline. Only original articles published from March 2020 to 31 August 2023 investigating the possible protective role of antiandrogens were included. In vitro or preclinical studies and reports not in the English language were excluded. The main objective was to investigate how antiandrogens may interfere with COVID-19 outcomes. Results: Among 1755 records, we selected 31 studies, the majority of which consisted of retrospective clinical data collections and of randomized clinical trials during the first and second wave of the COVID-19 pandemic. Conclusions: In conclusion, we can state that antiandrogens do not seem to protect individuals from SARS-CoV-2 infection and COVID-19 severity and, thus, their use should not be encouraged in this field.

## 1. Introduction

After emerging in the Chinese city of Wuhan in December 2019, the severe acute respiratory syndrome coronavirus 2 (SARS-CoV-2) rapidly spread across the globe, causing the Coronavirus disease 2019 (COVID-19) pandemic and affecting 764 million people with over 6.9 million deaths worldwide, up to the end of April 2023 [1,2]. SARS-CoV-2 infection can result in flu-like symptoms or, in some cases, in severe interstitial pneumonia leading to acute respiratory distress syndrome (ARDS) [3,4]. Epidemiological studies have demonstrated higher mortality among patients affected by pre-existing conditions such as hypertension, heart failure, diabetes, obesity, cancer and, in general, among men (adjusted hazard ratio (HR) of 1.59 (1.53–1.65)) [4,5].

At the molecular level, there is an increasing body of evidence that COVID-19 infection follows specific pathways, and the identification of SARS-CoV-2 entry mechanisms into host cells established the basis for the investigation of new therapies [6].

### 1.1. SARS-CoV-2 Structure

**Figure 1:**
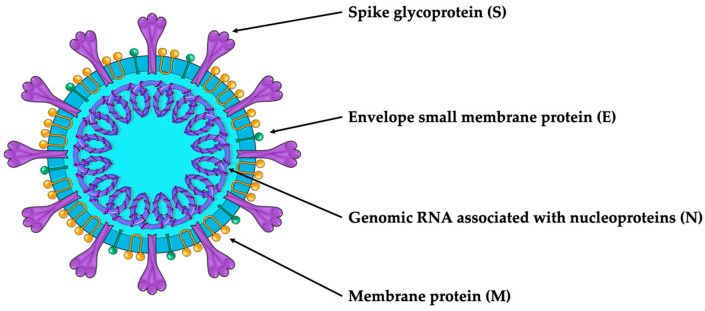
SARS-CoV-2 structure.

SARS-CoV-2 is an enveloped viral particle containing a single-stranded positive-sense RNA genome [7,8]. The virion comprises RNA associated with nucleoproteins (Ns) and membrane (M), spike (S) and envelope (E) capsid proteins involved in mediating host and viral membrane fusion [6] (Figure 1).

### 1.2. SARS-CoV-2 Internalization

**Figure 2:**
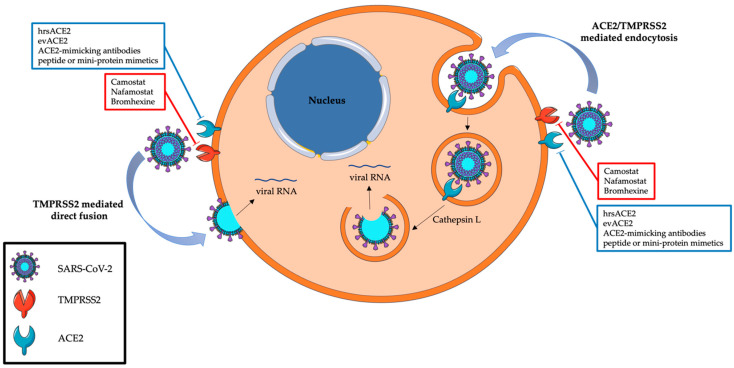
The internalization of SARS-CoV-2 and the best-known therapies targeting this mechanism. Red boxes contain a list of agents against TMPRSS2, blue boxes a list of therapies against ACE2.

Coronaviruses’ entry into host cells typically occurs in lung cells and may follow two routes: the endocytic or the non-endosomal/membrane fusion route. These processes are mediated by the cell surface molecule angiotensin-converting enzyme 2 (ACE2), which is recognized by the S viral protein for cell entry, and TMPRSS2, which cleaves and primes the S viral protein [9] (Figure 2). ACE2 is an enzyme involved in the regulation of the renin–angiotensin system and blood pressure by converting the vasoconstrictor angiotensin II to the vasodilator Ang-(1-7) [10,11,12,13,14,15,16,17]. TMPRSS2 is a serine protease whose gene transcription is regulated by testosterone and dihydrotestosterone through stimulation of the androgen receptor [14,18]; this protein is involved in different physiological and pathological processes, such as cancer and viral infections [9]. Studies on TMPRSS2 were mainly conducted in prostate cancer (PC) due to its high expression in prostate epithelium luminal cells: in this respect, cancer cells showed higher TMPRSS2 expression both in primary and advanced or metastatic PCs [19,20].

### 1.3. TMPRSS2-Targeting Therapies

Recently, TMPRSS2 inhibition has been described as an effective mechanism for the prevention of SARS-CoV-2 infection [21]. The first investigations regarded camostat mesylate, a serine protease inhibitor [21,22], tested in differentiated airway epithelial cells and distal airway organoids, finding a reduction in SARS-CoV-2 cell entry [23,24,25].

Besides the promising results obtained by in vitro studies, the pharmacological characteristics of camostat did not lead to it testing in clinical trials as rapidly metabolized and, thus, is not effective in humans [26]. A double-blind, randomized, placebo-controlled trial showed that camostat did not significantly improve patient clinical outcomes [27]. In addition, Kinoshita et al. reported how camostat did not substantially reduce the time to viral clearance compared with placebo for patients with mild to moderate infection [28].

Nevertheless, other TMPRSS2-targeted therapies have been tested, such as nafamostat mesylate and bromhexine. In vitro, Nafamostat resulted effective in preventing viral entrance [9,29,30,31]. Despite the efficacy achieved in a preclinical setting [30], animal and clinical studies are limited and some other works disconfirmed its efficacy [32,33]. However, the RACONA study (a prospective randomized, double-blind, placebo-controlled clinical trial investigating the efficacy and safety of nafamostat in COVID-19-hospitalized cases) highlighted some signals for a beneficial effect of this drug without serious adverse events [34]. Bromhexine is routinely used as a muco-active drug in upper respiratory tract infections; in this setting, given its activity on TMPRSS2, it was tested as an anti-COVID-19 drug [35]. In vitro studies confirmed its role in SARS-CoV-2 infection [36,37], but few and discordant data have been reported concerning bromhexine efficacy in clinical practice [38,39,40].

### 1.4. ACE2 and Spike Protein-Targeted Therapies

Considering the critical role of ACE2 in SARS-CoV-2 internalization by host cells, different therapies have been tested, such as human recombinant soluble ACE2 (hrsACE2) protein, ACE2-loaded extracellular vesicles (evACE2s), ACE2-mimicking antibodies/peptides and mini-protein mimetics [41]. Even if encouraging results of in vitro and phase I studies were reported on hrsACE2 and evACE2s, only ACE2-mimicking antibodies resulted in clinical relevance. Two of the most relevant ACE2 antibodies, P2C-1F11 and S2K146, showed promising inhibitory results in specific SARS-CoV-2 variants. P2C-1F11 showed results for the alpha, beta and gamma variants and S2K146 for the omicron variant [41,42,43]. The indirect targeting of ACE2 functions, through viral spike protein inhibition, has also been a reason for debate. Phase II clinical trials reported how the combination of bamlanivimab (also known as LY-CoV555 or LY3819253) with etesevimab (also known as LY-CoV016 or LY3832479), two monoclonal antibodies directed against SARS-CoV-2 spike protein, significantly decreased SARS-CoV-2 viral loads [44,45]. In addition, in a phase III clinical trial (NCT04497987), bamlanivimab plus etesevimab administration led to a more rapid decline in viral load and a 70% relative reduction in COVID-19-related hospitalizations or deaths [46]. 

### 1.5. Androgen Deprivation Therapy

Since the early phases of the SARS-CoV-2 pandemic, the higher prevalence of severe COVID-19 manifestations among men was evident. Of the several factors claimed to be responsible for this phenomenon, sex hormones seemed to play a prevalent role considering their activity as innate and adaptive immunity regulators [47,48,49,50,51,52]. Moreover, ACE2 and TMPRSS2 are regulated by androgens and are deeply involved in viral host cell entry. In this sense, antiandrogens (also referred to as androgen deprivation therapies, ADTs), such as antigonadotropins or 5-alpha reductase inhibitors (5-ARIs), have been considered as possible anti-SARS-CoV-2 therapies and have been investigated in both retrospective and prospective studies. Androgen deprivation can be achieved nowadays by long-acting luteinizing hormone-releasing hormone (LHRH) agonists or antagonists, also referred to as Gonadotropin Hormone-Releasing Hormone (GnHRH). Among the agonists, we can identify leuprolide, goserelin and triptorelin; degarelix, on the other hand, belongs to the LHRH antagonist group [53]. Antiandrogens (bicalutamide, nilutamide, flutamide) compete with androgens at the receptor level. They have been administered in the case of PC, although combined treatments with antiandrogens nowadays should only be considered if other treatments are not available. Over the years, several drugs changed the PC therapeutic scenario: second-generation androgen receptor targeting therapies (ARTAs) such as enzalutamide, abiraterone, apalutamide and darolutamide represented an innovative cornerstone of PC therapies [53]. Some of these have also been tested in the case of COVID-19 by exploiting their molecular mechanisms. Enzalutamide, a non-steroidal select antagonist of the androgen receptor, has been implicated in the reduction of TMPRSS2 levels in lung cells [54] and its role in COVID-19 treatment has been tested in a double-blinded, randomized phase II clinical trial [55]. Similarly, abiraterone acetate, a selective inhibitor of the enzyme 17α-hydroxylase/C17,20-lyase (CYP17) and a partial antagonist of AR, has been evaluated as an anti-SARS-CoV-2 therapy: in vitro results suggested that abiraterone might reduce the size of viral plaques and decrease N and S viral protein production [56]. Subsequent in vivo studies later disproved these results and abiraterone has consequently not been employed as an anti-viral treatment [57,58,59]. The effect of apalutamide, an oral competitive signaling inhibitor of the ligand-binding domain of the androgen receptor, on SARS-CoV-2 infection has also been tested by in vitro studies with Calu-3 lung adenocarcinoma cells and primary human nasal epithelial cells, finding a reduction in SARS-CoV-2 entry [6]. Proxalutamide is a novel third-generation antiandrogen that is currently under investigation for PC treatment. Compared to enzalutamide, it exerts higher anti-tumoral activity by simultaneously reducing androgen receptor levels and impairing lipid synthesis in PC cells, which has been linked to a further resistance mechanism [60,61]. Recent evidence claimed a prominent role of proxalutamide in treatment for COVID-19 patients, even if some concerns are still the subject of debate [62]. In addition to PC treatments, other drugs interfering with androgen activity have been investigated as anti-SARS-CoV-2 drugs. Among these, 5-alpha-reductase inhibitors (5-ARIs) should be mentioned. They act by impairing testosterone conversion into its more active form, dihydrotestosterone (DHT), by inhibiting 5-alpha reductase, a nuclear-bound steroid intracellular enzyme [63,64]. The most used 5-ARIs are finasteride and dutasteride [65], which have been largely used for benign prostate hyperplasia (BPH), androgen alopecia and hirsutism [66,67]. Considering their activities in interfering with androgens, and thus in ACE2 and TMPRSS2 activities, their role in preventing or limiting SARS-CoV-2 infection has been claimed in in vivo studies, finding encouraging results [68]. Table 1 offers an overview of the different treatments tested in this setting.

### 1.6. Other Anti-SARS-CoV-2 Treatments

In this complex therapeutic scenario, other drugs were concomitantly developed, leading to their use in clinical practice: dexamethasone, monoclonal antibodies and viral protease or polymerase inhibitors such as paxlovid, remdesivir and molnupiravir [69,70].

Despite the massive efforts to identify antiviral therapies, the greatest success in the fight against COVID-19 consisted in the development and distribution of effective SARS-CoV-2 vaccines: thanks to the vaccination campaign and the dramatic reduction in infections, on 5 May 2023, the World Health Organization (WHO) declared the end of COVID-19 as a public health emergency.

Although the global health emergency is now over, some uncertainties persist about the potential efficacy of hormone deprivation treatments against COVID-19. To offer a complete and up-to-date overview of this topic, we set up a systematic review collecting and evaluating all the current literature about the influence of antiandrogens in SARS-CoV-2 infection and COVID-19 outcomes in infected patients with or without PC.

## 2. Methods

### 2.1. Literature Search

A systematic literature review was performed according to the Preferred Reporting Items for Systematic reviews and Meta-Analysis (PRISMA) guidelines [71]. PUBMED and Medline were used as search engines, including results from January 2020 to August 2023. The search query line was ((ADT) OR (“androgen deprivation therapy” [All Fields]) OR (ARTA) OR (“AR target agent” [All Fields]) OR (“androgen receptor target agent” [All Fields]) OR (“androgen receptor signalling inhibitors” [All Fields]) OR (“AR signalling inhibitors” [All Fields]) OR (androgen) OR (ARSI) OR (Enzalutamide) OR (Abiraterone) OR (Apalutamide) OR (Darolutamide) OR (Proxalutamide) AND ((“SARS-CoV-2” [All Fields]) OR (“COVID-19” [All Fields]))).

### 2.2. Inclusion/Exclusion Criteria

We included only original articles, published from 1 March 2020 to 31 August 2023, showing the possible protective role of ADT associated or not with ARTAs or 5-ARIs. The exclusion criteria considered were as follows: case report studies, systematic or narrative reviews, editorials, guidelines and other studies not describing original data; in vitro or preclinical studies and non-English-language studies were excluded too. Peer-reviewed articles were considered as well and included.

The main objective was to investigate how antiandrogens may interfere with COVID-19 outcomes. The PICO framework items were as follows: P (population): positive SARS-CoV-2 patients affected or not by PC treated with ADT associated or not with ARTAs or treated with 5-ARIs; I (intervention group): ADT associated or not with ARTAs or 5-ARIs; C (control group): non-ADT patients; O (outcomes): SARS-CoV-2 or COVID-19 outcomes. A restriction on specific ADT was not considered, thus including all types of ADT, oral antiandrogens and 5-ARIs.

### 2.3. Data Extraction

Two authors (MC and MM) reviewed all study titles and abstracts, approving the selection according to the inclusion and exclusion criteria. Any disagreements about eligible and ineligible articles were resolved according to Delphi consensus criteria. First author, year, nation, sample size, data collection timepoints, quality assessment and outcomes were collected (Table 2). 

### 2.4. Quality Assessment

A quality evaluation, including risk of bias, was performed considering quality assessment tools for both observational cohort/cross-sectional studies and controlled intervention studies. Employed tools are available in Supplementary Materials (Supplementary S1). Two authors performed the quality assessment (MC and MM) and, in case of disagreement, a third author decided (RPM). Quality categories were as follows: good, fair and poor.

## 3. Results

**Figure 3:**
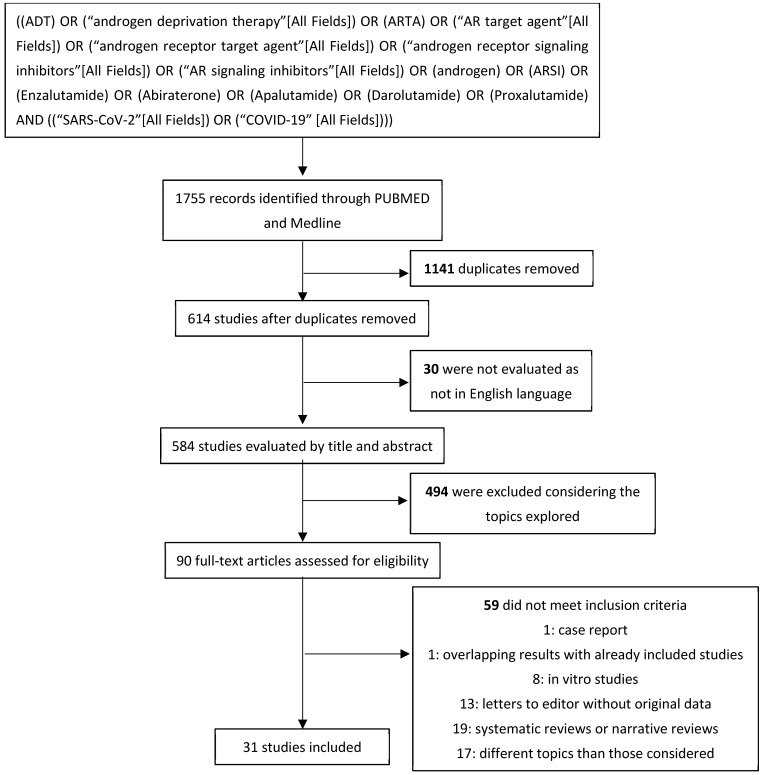
Systematic review’s flow diagram.

In total, 1755 studies were first recorded. Of these, 584 studies were then considered after excluding duplicates (n = 1141) and non-English-language articles (n = 30). After evaluating titles and corresponding abstracts, 494 were removed for exploring topics diverging from those considered. A total of 90 studies were extensively examined: 59 did not meet the inclusion criteria; 19 studies were excluded as narrative or systematic reviews; 17 were not included for being off-topic; 13 were editorials or letters to editors not describing original data; 8 studies reported results from in vitro studies; 1 study was not included for reporting overlapping results with other included analyses; and 1 case report was excluded. Thirty-one studies were then included in our systematic review after considering inclusion and exclusion criteria (Figure 3).

### 3.1. Characteristics of Included Studies

Table 2 summarizes all included studies (n = 31). Notably, the study by Welen et al. incorporated findings from both an RCT (COVIDENZA) and an epidemiological analysis. We addressed them separately in our work, considering them as distinct studies (n = 32) [55].

Fourteen studies considered patients affected by SARS-CoV-2 during the months of the COVID-19 pandemic (from March to July 2020); in seven studies, the monitoring period was extended just after July and until the end of 2020. In two studies [76,85], the monitoring period was not reported, and in all the others, data collection was extended until 2021. The majority of the studies investigated the role of ADT in mitigating SARS-CoV-2 sequelæ. Great heterogeneity in terms of sample size (42–26,508 patients, median = 514) and study design (17 out of 32 were retrospective studies, 9 prospective non-interventional studies and 6 RCTs) was identified. Eleven studies did not explicitly state the specific type or dosage of ADT prescribed. The different outcomes obtained in the studies included in our systematic review are sequentially reported in Table 2. The beneficial (“positive”) or detrimental (“negative”) effects of ADT on COVID-19 outcomes were further distinguished based on whether the effect was statistically significant (“association”) or not (“trend”). The absence of any correlation between ADT and COVID-19 outcomes was indicated with “No association”. Study quality assessments and the corresponding scores are summarized as well in Table 2.

### 3.2. Retrospective Studies

Concerning ADT among SARS-CoV-2 patients, Montopoli et al. were the first to report unexpected results. Considering data from 68 hospitals in the Italian region of Veneto, they included 4532 male patients with a SARS-CoV-2-positive polymerase chain reaction (PCR) test. Among these, 118 patients had a diagnosis of PC. Among 5273 prostate cancer patients already under ADT at the time of the infection, 4 developed SARS-CoV-2; in this subgroup, only 1 developed severe COVID-19 requiring hospitalization and ICU admission. Conversely, 31 patients in the non-ADT group developed severe disease, out of which 13 required ICU support and 18 died. Considering these results, the authors suggested a protective role of ADT in preventing severe forms of COVID-19, as subjects on ADT had a lower risk compared to non-ADT PC patients with an OR of 4.05 (95% CI: 1.55–10.59) [72].

Similar data were reported by Patel et al.: from March to June 2020 at Mount Sinai Health System, they considered 58 PC patients with COVID-19: of these, 22 were treated with ADT before SARS-CoV-2 infection. After adjusting for age, cardiac and pulmonary disease, the ADT group had a lower risk of hospitalization (OR 0.23, 95% CI: 0.06–0.79, *p* < 0.02). In this study, treatment with ADT appeared to be associated with a lower intubation rate (OR 0.31, 95% CI: 0.05–1.81, *p* = 0.192) and mortality (OR 0.37, 95% CI: 0.08–1.80, *p* = 0.22) [78], although neither of these differences were statistically significant.

A large observational American analysis, from March to September 2020, considered 26,508 consecutive SARS-CoV-2-positive veterans to study possible correlations between common medications and SARS-CoV-2 mortality. In total, 3197 patients (12%) had a diagnosis of cancer, and 1788 were treated with antiandrogens before SARS-CoV-2 infection. Such treatments were found to correlate with a reduced risk of COVID-19 mortality with an adjusted RR of 0.61 (95% CI: 0.51–0.73); these data were also confirmed in cases of an association between ADT and alpha-blockers, commonly prescribed as synergic drugs. Notably, in this study all concurrent medications were associated with a benefit in terms of SARS-CoV-2 outcomes. Although not entirely applicable to prostate cancer, this likely reflects the inclusion of healthier patients taking concurrent medications to control risk factors, such as blood pressure or diabetes, correlated with COVID-19 outcomes [75].

Similarly, between February and July 2020, Lee et al. collected data from a large cohort of male Veterans treated in the Veterans Health Administration, assessing the possible protective role of ADT as the primary endpoint among patients who tested positive for SARS-CoV-2. Of 25,006 patients who tested positive, 295 were under ADT at the time of viral infection (n = 186 for PC). Patients with PC and a second tumor were considered as well (n = 97). The comparison of PC patients on ADT to other cancer patients revealed that the use of ADT significantly correlated with a lower likelihood of SARS-CoV-2 infection (adjusted OR 0.88, 95% CI: 0.81–0.95, *p* = 0.001). Similarly, ADT use positively correlated with lower COVID-19 severity based on ICU admission, mechanical ventilation, or death (OR 0.72, 95% CI: 0.53–0.96; *p* = 0.03) [73].

However, contrasting results were obtained by other studies. Kwon and colleagues extracted data from five academic medical centers and 12 affiliated hospitals across California, from February to December 2020, thus including 5211 PC patients who tested positive for SARS-CoV-2. Of the 779 PC patients receiving ADT at the time of infection, 18 were found to be positive for SARS-CoV-2 (2.3%), while of the 4412 non-ADT PC patients, 79 tested positive (1.8%) with an OR of 1.30 (95% CI: 0.78–2.19, *p* = 0.31). Regarding mortality, 5.3% of men treated with ADT at the time of infection died compared to 9% who did not receive ADT (OR 0.56, 95% CI: 0.07–4.88, *p* = 0.6) [74].

An Italian real-world analysis, elaborated by Caffo and colleagues, included 1949 metastatic PC patients from 19 medical oncology departments, all treated with ADT combined or not with other treatments (ARTAs, chemotherapy or Radium-223). Of these, 36 had a diagnosis of SARS-CoV-2 infection (1.8%); most of them were hospitalized (61.1%) and 11 died due to COVID-19 [76]. Another concomitant multicenter retrospective analysis by Caffo et al. included 1433 mCRPC patients from 20 Italian oncological centers from February to June 2020. Of these, 34 (2.3%) tested positive for SARS-CoV-2, all under ADT. In total, 22 patients were hospitalized due to COVID-19 (64.7%) and 5 were admitted to the ICU; of all 34 positive patients, 13 died [77]. In conclusion, both of Caffo’s analyses do not show a benefit of ADT in terms of SARS-CoV-2 infection rates and mortality.

Koskinen and colleagues, between March and May 2020, carried out a retrospective cohort study based on PC patient records from the Hospital District of Helsinki and Uusimaa in Finland. They enrolled 352 men (134 of them treated with ADT). All were tested for SARS-CoV-2: 17 had positive results for SARS-CoV-2, with 6 of them being treated with ADT at the time of infection. Remarkably, in such series, ADT also included flutamide and bicalutamide, which are seldom used as types of ADT. These data disproved the protective role of ADT against SARS-CoV-2 infection as the frequency of testing positive was not associated with ADT (OR 0.88; 95% CI: 0.32–2.44, *p* = 0.81). Regarding the occurrence of death or the need for intensive care, these did not differ in the two groups with an OR of 0.53 (95% CI: 0.04–6.66, *p* = 0.63) [57].

In a large Swedish study, Gedeborg and colleagues investigated the prevalence of SARS-CoV-2 infection and subsequent hospitalization among 114,547 men affected by PC. Data were extracted from the Swedish Prostate cancer database and patients were followed from February 2020 to December 2020. A total of 1695 patients tested positive for COVID-19, 596 of whom were under ADT (even flutamide and bicalutamide) at the time of infection. After adjusting for age, comorbidity and PC risk category, the HR for testing positive for COVID-19 for the ADT population was in 1.3 (95% CI: 1.1–1.5). Considering the risk of subsequent hospital admission or death, these results were even higher (OR, 1.4; 95% CI: 1.0–1.9). Again, these data seem to disprove the protective role of ADT in preventing SARS-CoV-2 infection and its sequelae, enforcing data from another smaller Swedish case–control analysis [58,86].

COVIDENZA authors (part 3 of the study) also aimed to investigate the impact of antiandrogens on SARS-CoV-2 infection in a retrospective cohort of PC patients. They included 7894 PC patients affected by SARS-CoV-2 from Swedish national registers. Patients were categorized into four groups: those under a single antiandrogen treatment (n = 676), those who underwent surgical or chemical castration with ADT (n = 798), patients undergoing combined therapy with ADT and abiraterone or enzalutamide (n = 214) and patients with no ongoing or later hormonal therapy (n = 6206). The main outcomes considered were the need for hospitalization, ICU admission and death. After adjusting for age and comorbidities, the use of ADT plus abiraterone or enzalutamide was associated with higher mortality, with an odds ratio of 2.03 (95% CI: 1.37–3.00, *p* < 0.001) [55].

Jimenez-Alcaide et al.’s, Shah and colleagues’, Dalla Volta and others’, and Schmidt et al.’s studies were closely aligned with those previously discussed, as they failed to identify statistically significant benefits in terms of SARS-CoV-2 outcomes for patients undergoing ADT. Therefore, these studies are not described in detail. For further information, Table 2 presents all the necessary elements for interpreting the analyses [79,80,82,89].

### 3.3. Prospective Observational Studies

In addition to retrospective studies, prospective observational studies have been conducted since 2020. Among these, a prospective Turkish study, between August 2020 and June 2021, enrolled 365 PC patients, 138 of whom were being treated with ADT. A total of 43 patients tested positive for SARS-CoV-2; 13 were concomitantly treated with ADT. In this report, the infection rate for ADT PC patients was similar to that of the control group (9.4% vs. 13.2%, *p* = 0.275). No mortality or ICU admissions were detected and hospitalization rates were similar in both groups (2.9% vs. 0.9%, *p* = 0.205). The authors re-evaluated the risk and severity of COVID-19 by excluding patients with COPD, but in this case, too, no difference was found between the two groups (12.6% vs. 9.0%, *p* = 0.316; 0.5% vs. 2.5%, *p* = 0.105, respectively) [87].

A prospective registry of all patients tested for SARS-CoV-2 was evaluated by Klein et al. from March to June 2020, enrolling 1779 PC patients, 102 of whom tested positive for SARS-CoV-2; among the patients already under ADT, 5.6% had positive results for SARS-CoV-2, compared to 5.8% of the non-ADT PC patients. In this regard, a multivariable analysis did not show a difference in infection risk for PC patients treated with ADT (OR 0.93, 95% CI 0.54–1.61, *p* = 0.8) [83].

Some studies also considered PC patients treated with ADT and concomitantly with abiraterone or enzalutamide at the time of SARS-CoV-2 infection. A study by Unlu and colleagues, which included 25 patients, of whom 11 were treated with abiraterone, enzalutamide or bicalutamide, did not find any significant difference in terms of hospital stay, ICU admission rate, intubation percentage and mortality, even if the results were numerically lower in the ADT group compared to non-ADT patients [59]. Similar results were achieved by Davidsson and colleagues: In this study evaluating 655 PC patients, 224 of whom were undergoing ADT, a comparative group comprising patients diagnosed with benign prostatic hyperplasia was also taken into consideration (n = 240 patients). The specific treatments administered to this latter subgroup remain undefined, thereby precluding the exclusion of exposure to 5-alpha reductase inhibitors, which are also potentially implicated in modulating SARS-CoV-2 infection. With the primary endpoint being the presence of antibodies against SARS-CoV-2 and self-reported symptoms compatible with COVID-19 serving as secondary outcomes, no statistically significant difference was discerned between the two groups [90].

### 3.4. Randomized Clinical Trials

To elucidate the potential correlation between an assumed protective role of ADT against SARS-CoV-2 infection, several randomized clinical trials were conducted.

In this sense, interesting results were obtained by Nickols et al. Based on the hypothesis that temporary treatment with ADT might benefit COVID-19 patients, a phase II placebo-controlled, double-blind, randomized clinical trial was conducted to test the efficacy of degarelix. From July 2020 to April 2021, 96 patients were enrolled at the time of hospital admission, 64 of whom were treated with degarelix. The primary endpoints were mortality reduction, an ongoing need for hospitalization and a requirement for mechanical ventilation on day 15 after randomization. In a planned interim analysis, no statistically significant differences were identified between the degarelix and placebo groups in terms of mortality, hospitalization or the need for mechanical oxygen support. These results led to the trial’s discontinuation [92].

As stated above, other analyses investigated the role of specific antiandrogen treatments like enzalutamide. In a randomized phase II clinical trial [55], SARS-CoV-2 patients were randomized 2:1 to receive 5 days of oral enzalutamide plus SOC versus SOC alone. The inclusion criteria comprised positivity on a SARS-CoV-2 PCR test, being older than 50 years old and not requiring mechanical ventilation. An ongoing hormonal cancer therapy, an immunosuppressive disease or other critical illness were considered exclusion criteria. From June to November 2020, 42 patients were included. The primary outcome was the time to mechanical ventilation (or death) and time to hospital discharge, whichever came first. Two patients in the enzalutamide arm and one in the control group reached the primary outcome requiring mechanical ventilation. At the time of hospital discharge, among the patients followed at least for 1 week, the median stay at the hospital was 9 days for the enzalutamide arm and 6 days in the control group; in this sense, the adjusted age and gender HR for discharge from the hospital was 0.43 (95% CI: 0.20–0.93, *p* = 0.032). Of note, patients in the enzalutamide arm required a longer time on supplemental oxygen, with a median difference of 4 days (MAD 5.9; *p* = 0.022). Furthermore, SARS-CoV-2 viral loads measured by PCR between the two arms did not differ significantly, with a difference in ΔCt of −5.6 on day 4 (95% CI: −10.7 to 0.8, *p* = 0.084) [55].

Different results were reported by Cadegiani et al. in the randomized, double-blinded, placebo-controlled, multiregional clinical trial Proxa-Rescue AndroCoV. This trial included patients with an age above 18 years who tested positive for SARS-CoV-2 by PCR test and who needed hospitalization in the previous seven days. Patients who required mechanical ventilation; who had liver damage (AST > 250 U/L), kidney injury (serum creatinine > 2.5 mg/mL or calculated eGFR below 30 mL/min) or class III or IV congestive heart failure; and those who were pregnant, breastfeeding or planning to conceive within 90 days after randomization were excluded. Similarly, the use of immunosuppressive agents or antiandrogens was not permitted. Patients were randomized to receive proxalutamide 300 mg/day plus the standard of care vs. placebo plus the standard of care for 14 days. The primary outcome was the recovery rate 14 days after randomization based on scores 1 and 2 on the eight-point COVID-19 ordinal scale, or the proportion of patients discharged from the hospital alive before 14 days of treatment. Secondary outcomes included the recovery rate (scores 1 and 2) on day 28, the all-cause mortality rate (score 8) on day 14 and on day 28 and hospitalization stay and time-to-discharge alive after randomization. The joint analysis reported data for both arms from the North and South Brazilian regions. Overall, 778 subjects were enrolled: of these, 423 were treated with proxalutamide. Patients in the proxalutamide arm achieved better outcomes: the recovery rate over 14 days after randomization was 121% higher than that for the control group (recovery ratio 2.21, 95% CI: 1.92–2.56); these results were also confirmed considering the 28-day recovery rate, which was 81% higher in the proxalutamide vs. the placebo group. In the proxalutamide arm, the all-cause mortality rate on day 14 was 8.0% vs. 38.9% in the placebo arm, with a risk ratio (RR) of 0.21 (95% CI: 0.15–0.30); also, in this case, the trend was confirmed at 28 days, with an RR of 0.22 (95% CI: 0.16–0.30). Hospitalization was also shorter in the proxalutamide arm: median 8 days (IQR: 6–13) vs. 12 days (IQR: 8–18) (*p* < 0.0001) [91].

Proxalutamide (200 mg/day) was also tested in newly diagnosed male COVID-19 patients compared to a placebo in a double-blinded RCT to evaluate the 30-day hospitalization rate. Compared to placebo the 30-day hospitalization rate resulted 2.2% vs. 26% of placebo, *p* < 0.001 with a RR of 0.09 (95%CI: 0.03–0.27), thus showing a benefit of proxalutamide in newly diagnosed COVID-19 patients [97].

Notably, these results were questioned soon after their release: some controversies regarding the inadequacy of the study’s conclusions were claimed based on the methodology of the study. In detail, the process of treatment and control allocation was not sufficiently random, thus leading the *Frontiers* Editorial Office to retract the study [98]. Cadegiani’s results regarding the The Proxa-Rescue AndroCoV trial were also questioned and, on 15 October 2021, the Brazilian National Health Council published a press release regarding some irregularities in the proxalutamide clinical trial, informing of subsequent legal actions [99,100].

### 3.5. 5-ARIs

Considering the more severe manifestation of COVID-19 among men affected by androgen alopecia (AGA) [101,102] and the preliminary evidence of a lower infection rate among those treated with 5-ARIs for AGA, this subset of patients was investigated. In this regard, McCoy and colleagues, in a retrospective cohort analysis, included 300 SARS-CoV-2 patients afferent to a Brazilian dermatological clinic affected by AGA, treated or not with 5-ARIs. Among SARS-CoV-2-positive patients, investigators evaluated the prevalence of COVID-19-related symptoms. Forty-eight of these patients were treated with 5-ARIs (dutasteride 0.5 mg/daily dose) for at least six months; these patients demonstrated a lower frequency of COVID-19 symptoms compared to patients not treated with 5-ARIs with a statistically significant difference (*p* < 0.05). The major differences regarded symptoms like anosmia, ageusia, headache and dry cough [93].

An Italian cohort analysis among patients admitted to “Istituto Clinico Humanitas Gavezzani hospitals of Milan and Bergamo” due to SARS-CoV-2 infection achieved similar results. Among 1432 patients admitted from 1 March to 24 April 2020, 45 patients were concomitantly treated with 5-ARIs (dutasteride, finasteride) for BPH for at least six months. No effect of 5-ARIs was found considering ICU admission and death: HR = 0.79 (95% CI: 0.54–1.15, *p* = 0.22) and OR = 1.23 (95% CI: 0.81–1.87, *p* = 0.33). In this cohort of patients admitted to the hospital due to COVID-19, the fraction of men on 5-ARIs was lower than that in an age-matched control population (5.57 vs. 8.14%, *p* = 0.0083, 95% CI: 0.75–3.97%), possibly indicating that 5-ARIs protect one from COVID-19 symptoms requiring hospital care [94].

Another prospective cohort study (NCT04368897) was conducted in this regard. Patients with SARS-CoV-2 infection were categorized based on the use of antiandrogens or spironolactone (which exhibits significant antimineralocorticoid and moderate antiandrogenic activity, along with a mild inhibitory effect on steroidogenesis) for at least six months before hospitalization. Out of the 77 enrolled patients, 12 were taking 5-ARIs (finasteride, n = 2; dutasteride, n = 9) or spironolactone (n = 1). In this group, a higher age (80.6 ± 8.2 vs. 66.4 ± 12.2, *p* = 0.0002) and a lower rate of ICU admissions were observed (1/12 (8%) vs. 38/65 (58%), *p* = 0.0015), thus confirming the primary endpoint of the study. Even when considering an age-matched analysis, the percentage of patients admitted to the ICU among those taking 5-ARIs or spironolactone was significantly lower compared to those who did not take them (1/12 (8%) vs. 17/36 (47%), *p* = 0.018). The RR for ICU admission in subjects taking 5-ARIs or spironolactone compared to the age-matched group was 0.19 (95% CI: 0.03–1.28). After excluding the patient using spironolactone (prescribed for cardiovascular reasons) from the analysis, the statistical significance of 5-ARIs in reducing ICU admissions persisted (*p* = 0.0028) [88].

In order to verify the potential protective role of dutasteride and verify previous data, a double-blinded, placebo-controlled clinical trial was initiated. Patients were thus enrolled in the Early Antiandrogen Therapy with dutasteride for the COVID-19 trial (EAT-DUTA AndroCoV Trial) from June to October 2020. Patients were randomized to dutasteride 0.5 mg daily or placebo for 30 days or until fully recovery from COVID-19. Furthermore, all patients received the standard of care based on nitazoxanide 500 mg twice daily for six days and azithromycin 500 mg/day for five days. The time to remission; oxygen saturation (%); positivity rate of PCR-SARS-CoV-2; and biochemical analysis, such as ultrasensitive C-reactive protein, D-dimer, lactate, lactate dehydrogenase (LDH), erythrocyte sedimentation rate (ESR), ultrasensitive troponin and ferritin were considered the study’s outcomes. Patients in the experimental arm were found to have a lower time for infection remission (9.2 ± 4.3 days vs. 16.3 ± 8.3 in the placebo group, *p* < 0.001). A similar trend was also observed for fatigue remission (5.5 ± 3.2 days vs. 10.3 ± 8.4 days, *p* < 0.001) and anosmia (5.6 ± 4.0 days vs. 11.1 ± 6.6 days, *p* < 0.001). Notably, on day seven, 64.7% of patients included in the dutasteride group and 11.8% of men from the placebo group had undetectable nasopharyngeal SARS-CoV-2 virus or viral fragments (*p* = 0.0094). This trend was also confirmed on day 14: in total, 88.3% of the dutasteride group and 54.2% of the placebo group achieved non-detectable SARS-CoV-2. A relevant point regarding the study design concerns the administration of the experimental drug in an early stage of SARS-CoV-2 infection in patients not needing hospitalization. Similarly, the authors did not explore the influence of both hydroxychloroquine (four patients treated in the placebo and three in the dutasteride group) and Ivermectin (six patients treated in both arms) [96].

Otherwise, in another study, by enrolling 80 hospitalized COVID-19 patients aged >50 years old, after randomization to 5 mg of finasteride plus SOC vs. SOC alone, no significant differences were found in terms of mortality (2.5% vs. 10%, *p* = 0.166) and duration of hospital stay for patients treated with finasteride [95].

## 4. Discussion

On 5 May 2023, the WHO declared the end of the COVID-19 pandemic as a major global health emergency [103], but understanding whether some therapies may protect individuals from infection or from adverse outcomes could prove useful in the case of future relapses or future outbreaks of novel coronaviruses. In particular, some prognostic factors and therapeutic implications remained unsolved. Many therapeutic attempts have been made due to the severity of COVID-19 and the heavy burden inflicted by the pandemic on health systems. In addition to the urgent need for effective anti-viral treatments, the demand for preventive strategies such as vaccines became a priority, which led to the clinical testing and approval of several vaccines within about a year, thanks to whom COVID-19 was efficiently contrasted. Although such a global “tsunami” dramatically overwhelmed our lives, thus changing our habits, some important lessons have been learned. One of them surely regards the great impact of the mRNA vaccines that showed efficacy and a good safety profile. Another relevant lesson regarded the need for a fast drug development system, which led to the hypothesis of repurposing already-approved drugs starting from viral molecular mechanisms and epidemiological data. In this sense, great attention has been given since the first phases of the COVID-19 pandemic to the higher clinical severity after SARS-CoV-2 infection among men compared to females, despite equivalent infection rates [104,105]. Different hypotheses have been formulated regarding the potential contributions of concomitant conditions, such as the higher percentage of tobacco smoking among men [106,107], estrogens’ immunologic modulating effects [108] and total testosterone levels [109,110,111].

In detail, estrogens exhibit a dual effect depending on their concentration levels. At lower doses resembling those found in post-menopausal women, they display an immune-stimulatory impact, prompting the differentiation of inflammatory dendritic cells, heightened production of IL-4 and IFNα and an increased Th1-type and cell-mediated responses. In contrast, at higher doses typical in premenopausal women, estrogens foster anti-inflammatory Th2 responses and inhibit the pro-inflammatory innate immune response [47].

Similarly, testosterone contributes to regulating the immune response by modulating the expression of IL-6, a mediator of the acute phase response. To explore this aspect, Rastrelli and colleagues enrolled 31 male patients with SARS-CoV-2, evaluating how total testosterone (TT) and calculated free testosterone (cFT) impact ICU admission or death. Patients with TT < 5 nmol/L or cFT < 100 pmol/L were at a higher risk for ICU admission and mortality, with a respective increase of 20–30-fold and 10–15-fold [112]. Other case–control studies have also supported these findings [113], and a possible state of transient primary hypogonadism has been suggested, related to the severity of illness. This may be associated with damage to the testis’ epithelium by SARS-CoV-2 due to the high expression of TMPRSS2 and ACE2 in spermatogonia, Sertoli and Leydig cells, as evidenced by a decreased T:LH ratio [114,115,116].

With regard to the role of ADT, the study by Montopoli et al. and its encouraging findings have catalyzed the development of analogous investigations, thus immediately kindling the scientific discourse. Consequently, various studies were undertaken, albeit predominantly of a retrospective nature, as demonstrated by the results of our systematic review: out of 32 studies, 17 were structured as retrospective studies and, among these, 13 did not yield positive outcomes. The quality assessment was found to be evenly balanced for both negative and positive studies. Among the conducted studies, prospective observational studies assumed a certain significance. Among these (n = 9), the majority (n = 7) did not yield positive outcomes, and once again, the quality assessment exhibited a well-balanced distribution.

Furthermore, within the same setting, randomized trials were conducted, totaling six in our series. Among these, three did not demonstrate any advantages associated with the administration of ADT or similar interventions. These results should be interpreted in light of both the conducted quality assessment (two out of three positive studies achieved a “poor” quality score) and the circumstances surrounding the trials in which proxalutamide was tested, thus leading to subsequent controversies [98].

As many of the studies reported conflicting, controversial results, several different systematic reviews and meta-analyses have been conducted, albeit without finding univocal evidence [117,118,119,120]. As an example, Motlagh et al., by including five studies, did not find any association between ADT use and SARS-CoV-2 infection or COVID-19 outcomes in PC patients [120]. Despite these results, different meta-analyses also did not unequivocally find an increased risk for COVID-19 in PC patients using these drugs, thus not requiring their discontinuation in case of infection [117,119,120].

Considering the different results of the included studies and our analysis, even characterized by its limits, it is not possible to surely assess a role of antiandrogens either in preventing SARS-CoV-2 infection or in limiting its course.

### 4.1. Limits and Controversies

One of the limits of our systematic review regards the high heterogeneity of the described findings that simply reflect the inherent diversity of the included studies in terms of numerosity, inclusion criteria and the characteristics of enrolled patients. For instance, Hunt and colleagues, in their retrospective analysis, included the largest cohort of patients (26,508) among those considered in our series, while seven studies (two of them retrospective, two prospective and three RCTs) enrolled less than 100 patients.

Another critical aspect regards the different kinds of patients enrolled, and this may justify the different findings of Montopoli’s and Caffo’s analyses. Montopoli’s analysis included patients with a PC diagnosis without specifying the disease stage and the reason for ADT assumption. On the other hand, Caffo et al. included mHSPC/mCRPC in the first report and only mCRPC in the second analysis: in this sense, the advanced disease stage may overshadow any potential benefit of ADT for PC patients with COVID-19.

Considering Montopoli’s report’s relevance, different works tried to interpret these results. In this regard, O’Callaghan et al. calculated in 434 the number of patients needed to be treated with ADT for the prevention of one case of COVID-19 [121], thus reducing the magnitude of the work.

Another limit of some of the included studies regards the inconsistencies and lack of information about the type and dosage of ADT administration.

A further possible controversy consists in the timing of antiandrogen administration: considering the mechanisms of antiandrogens in blocking viral entry through TMPRSS2 and ACE-2 inhibition, their primary effect in preventing SARS-CoV-2 infection and disease progression should be achieved with the early administration of antiandrogens.

Another limit regards the data paucity on SARS-CoV-2 variants that influence infectiousness and COVID-19 severity. Vaccination status was also not defined, even though many studies collected data in 2020 when COVID-19 vaccines were still under investigation. Furthermore, the findings of the surveyed studies may be influenced by the higher percentage of comorbidities among ADT patients and old age, which may mask any protective role of ADT [57,81,117]. ADT-related side effects should also be taken into account, as higher cardiovascular risk, reduced muscle mass, higher sedentariness and impaired lipid levels might all have an impact on COVID-19 outcomes.

ICU admission was considered a potential endpoint across various studies to assess the protective effects of the drugs under investigation. Notably, this outcome may be influenced by additional factors, including patients’ age, comorbidities, performance status and clinical prognosis. Unfortunately, these aspects were not taken into account in the analyses included in our series, thereby representing a potential limitation.

As androgen inhibitors, 5-ARIs were also investigated. In this setting, a selection bias cannot be completely ruled out, as 5-ARIs are usually administered to patients with good performance status and who are not affected by severe comorbidities. Therefore, more conclusive evidence from RCTs is needed to conclusively evaluate any potential protective role of 5-ARIs.

### 4.2. Future Perspectives

Many topics still need to be thoroughly investigated in SARS-CoV-2 pathogenesis; in this sense, genetic features (e.g., the influence of P1245 polymorphic variants of HSD3B1 genes on COVID-19 severity) may contribute to explain the heterogeneous disease course of COVID-19 in different patients and more studies should therefore be encouraged in this direction [122]. The ethical implications of administering antiandrogens like degarelix or enzalutamide to non-cancer patients, even those affected by SARS-CoV-2 infection, warrant discussion concerning potentially relevant side effects. Conversely, the use of 5-ARIs may be more feasible in terms of side effects, suggesting a possible future direction of investigation.

## 5. Conclusions

The COVID-19 pandemic has represented a major health and societal concern in recent years. In this context, public vaccination campaigns represented a turning point in facing the pandemic. In the same scenario, different therapies have been tested and, among these, there was great debate regarding the role of antiandrogens in interfering with SARS-CoV-2 infection and mitigating COVID-19 severity. In spite of the numerous studies that were conducted, divergent, and thus not conclusive, results were obtained. Our work tried to summarize the current literature evidence, but the high heterogeneity and the paucity of RCTs did not lead to meaningful conclusions regarding a beneficial role of antiandrogens in COVID-19. Therefore, the use of antiandrogens should not be encouraged for SARS-CoV-2 infection prevention and COVID-19 treatment.

## Figures and Tables

**Table 1:** Antiandrogen treatments.

Drug	Molecular Mechanism	PC Treatment Setting
**Leuprolide**	LHRH analogues: lead to eventual down-regulation of LHRH receptors, thus suppressing LH and FSH production and, subsequently, testosterone synthesis after an initial flare-up	nmHSPC mHSPC nmCRPC mCRPC
**Triptorelin**		
**Goserelin**		
**Degarelix**	LHRH-antagonists: by binding to LHRH receptors, lead to a rapid decrease in FSH and LH production and then testosterone synthesis	
**Bicalutamide**	Androgen receptor inhibitor	nmHSPC mHSPC nmCRPC mCRPC
**Flutamide**		
**Nilutamide**		
**Enzalutamide**	Androgen receptor inhibitor	nmCRPC (PSA-DT < 10 months) mHSPC mCRPC
**Abiraterone**	Selective, irreversible CYP17inhibitor. Interference with androgen biosynthesis. Partial AR antagonist	High-risk LA-PC
		mHSPC mCRPC
**Apalutamide**	Androgen receptor inhibitor	nmCRPC (PSA-DT < 10 months) mHSPC
**Darolutamide**	Androgen receptor inhibitor	nmCRPC (PSA-DT < 10 months)
**Proxalutamide**	Third-generation AR receptor inhibitor	Currently under investigation
**Finasteride**	5-alpha-reductase inhibitors: impair the conversion of testosterone into dihydrotestosterone	Male alopecia, benign prostate hyperplasia
**Dutasteride**		

LHRH: luteinizing hormone-releasing hormone; nmHSPC: non-metastatic hormone-sensitive prostate cancer; mHSPC: metastatic hormone-sensitive prostate cancer; nmHSPC: non-metastatic castration-resistant prostate cancer; mHSPC: metastatic castration-resistant prostate cancer; FSH: follicle-stimulating hormone; LH: luteinizing hormone; PSA-DT: prostate specific antigen doubling time; LA-PC: locally advanced prostate cancer; CYP17: cytochrome P450 17α−hydroxy/17,20-lyase.

**Table 2:** Characteristics of studies included.

Authors	Year *	Country	Design	Sample Size	Time of Data Collection	Outcomes 1 Ep	Drug	Quality-Bias Assessment	Statistical Evaluations
**Retrospective studies**									
**Montopoli** **et al.** **[72]**	2020	Italy	Retrospective cohort	4532 men, 118 PC pts	Last update on April 2020	ADT impact on COVID-19 clinical outcomes	ADT (NOS)	Fair	Positive association
**Lee** **et al.** **[73]**	2020	USA	Retrospective cohort	25,006 SARS-CoV-2 pts, 95 under ADT	February–July 2020	ADT impact on COVID-19 clinical outcomes	ADT (NOS)	Good	Positive association
**Koskinen** **et al.** **[57]**	2020	Finland	Retrospective cohort	352 PC pts, 134 under ADT	March–May 2020	ADT impact on COVID-19 clinical outcomes	History of orchiectomy, GnRH ag./antag. Ars, abiraterone	Fair	No association
**Kwon** **et al.** **[74]**	2020	USA	Retrospective cohort	5211 PC pts, 799 under ADT	February–December 2020	ADT impact on COVID-19 clinical outcomes	GnRH ag./antag.	Good	No association
**Hunt** **et al.** **[75]**	2020	USA	Retrospective cohort	26,508 pts (23,659 men)	March to September 2020	30-day mortality from first SARS-CoV-2-positive test	Concomitant therapies (including ADT)	Good	Positive trend
**Caffo** **et al.** **[76]**	2020	Italy	Retrospective cohort	1949 mCRPC pts	2020, NOS	SARS-CoV-2 diffusion among PC patients	ADT for mCRPC	Fair	Negative trend
**Caffo** **et al.** **[77]**	2020	Italy	Retrospective cohort	1443 mCRPC	February–June 2020	Death rate and possible impact of the PC therapy/history on mortality	ADT for mCRPC	Good	No association
**Patel** **et al.** **[78]**	2020	USA	Retrospective cohort	58 PC pts, 22 under ADT	March–June 2020	ADT impact on COVID-19 clinical outcomes	GnRH ag./antag. (within 3 months) or [testosterone] < 50 ng/dL within 6 months	Fair	Positive trend
**Alcaide** **et al.** **[79]**	2021	Spain	Retrospective cohort	1349 PC pts, 156 under ADT	March–May 2020	ADT impact on COVID-19 clinical outcomes	ADT (NOS)	Good	No association
**Schmidt** **et al.** **[80]**	2021	USA	Retrospective cohort	1106 PC pts	March–February 2021	30-day mortality	ADT for at least 6 months	Good	No association
**Duarte** **et al.** **[81]**	2021	Brazil	Retrospective cohort	199 PC pts, 156 under ADT	2020 to April 2021	Mortality	ADT (NOS)	Good	No association
**Welen et al.** **[55]**	2021	Sweden	Retrospective cohort	7894 PC pts, 4 GPs. GP3, n = 214 (ADT plus abiraterone/enzalutamide)	February 2020 to May 2021	ADT impact on COVID-19 clinical outcomes	ADT, abiraterone, enzalutamide	Good	Negative association for GP3
**Gedeborg** **et al.** **[58]**	2022	Sweden	Retrospective cohort	1695 SARS-CoV-2 PC pts, 596 under ADT	February–December 2020	ADT impact on COVID-19 clinical outcomes	Bicalutamide, Flutamide, GnRH ag., abiraterone, enzalutamide	Good	Negative association
**Unlu** **et al.** **[59]**	2022	USA	Retrospective cohort	146 PC pts, 25 under ADT	March–October 2020	ADT impact on COVID-19 clinical outcomes	GnRH ag./antag., AR antag., enzalutamide, abiraterone	Good	Positive trend
**Dalla Volta** **et al.** **[82]**	2022	Italy	Retrospective cohort	83 PC pts under ADT	February–April 2020	ADT impact on COVID-19 clinical outcomes	ADT (NOS)	Fair	No association
**Prospective observational studies**									
**Klein** **et al.** **[83]**	2020	USA	Prospective registry cohort study	1779 PC pts, 304 under ADT	March–June 2020	SARS-CoV-2 infection rate, disease severity	ADT (NOS)	Good	No association
**Di Lorenzo** **et al.** **[84]**	2020	Italy	Prospective obs. cohort	72 PC pts	March 2020	ADT impact on COVID-19 clinical outcomes	LHRH ag./antag. abiraterone, enzalutamide, apalutamide	Fair	Positive trend
**Ianhez** **et al.** **[85]**	2020	Brazil	Snowball sampling population survey	554 healthy and 17 COVID-19 volunteers under AR	Not reported	ADT impact on COVID-19 clinical outcomes	ADT for PC, 5-ARIs, bicalutamide, spironolactone and cyproaterone	Fair	No association
**Gedeborg** **et al.** **[86]**	2021	Sweden	Population-based nested case—control cohort	474 SARS-CoV-2 PC pts	March–December 2020	ADT impact on COVID-19 clinical outcomes	Bicalutamide, flutamide, GnRH ag., abiraterone, enzalutamide	Good	Negative association
**Kazan** **et al.** **[87]**	2021	Turkey	Obs. prospective cohort	365 PC pts, 138 under ADT	August–June 2021	ADT impact on COVID-19 clinical outcomes	ADT (NOS)	Good	No association
**Goren** **et al.** **[88]**	2021	Spain	Prospective cohort	77 pts, 12 under Ars	March–May 2020	ICU admission rate	Antiandrogens for at least 6 months	Fair	Positive association
**Shah** **et al.** **[89]**	2022	USA	Multi institutional obs. study	465 PC pts, 148 receiving ADT	March 2020	Overall survival	ADT (NOS)	Good	No association
**Davidsson** **et al.** **[90]**	2023	Sweden	Prospective cohort	655 PC pts, 224 undergoing ADT; 240 patients with BPH	April–early 2021	COVID-19 outcomes and positive SARS-CoV-2 antibody serology	ADT, bicalutamide, abiraterone, enzalutamide	Good	No association
**Randomized clinical trials**									
**Welen** **et al.** **[55]**	2021	Sweden	RCT	42 COVID-19 pts	July–November 2020	Time to mechanical ventilation (or death)/hospital discharge	Enzalutamide	Good	No association
**Cadegiani** **et al.** **[62]**	2021	Brazil	RCT	236 volunteers: 171 (proxalutamide arm); 65 (placebo arm)	2021 (NOS)	Percentage of negative pts negative for SARS-CoV-2 on day 7	Proxalutamide	Poor	Positive association
**Cadegiani** **et al.** **[91]**	2021	Brazil	Joint analysis of RCT	778 volunteers; 423 in the proxalutamide arm	2021 (NOS)	14-day and 28-day recovery	Proxalutamide vs. placebo in addition to SOC	Poor	Positive association
**Nickols** **et al.** **[92]**	2022	USA	RCT	96 pts: 62 experimental vs. 34 placebo group	July–April 2021	Mortality, need for hospitalization, need for mechanical ventilation on day 15	Degarelix 240 mg s.c. vs. saline placebo	Good	No association
**5-ARIs**									
**McCoy** **et al.** **[93]**	2020	Brazil	Retrospective cohort	300 SARS-CoV-2 pts, 48 under dutasteride	June–July 2020	COVID-19 severity among men under 5-ARIs	Dutasteride for at least 6 months	Fair	Positive association
**Lyon** **et al.** **[68]**	2021	USA	Retrospective cohort	1888 people, 944 5-ARI users	March–February 2021	SARS-CoV-2-positivity rate	5-ARIs (finasteride and dutasteride)	Good	Positive association
**Lazzeri** **et al.** **[94]**	2022	Italy	Obs. case–control cohort	943 pts, 43 under 5-ARIs	March–April 2020	Impact of 5-ARIs on COVID-19 clinical outcomes	5-ARIs (finasteride, dutasteride) for at least 6 months	Fair	Positive association (for men aged >55)
**Zarehoseinzade et al.** **[95]**	2021	Iran	Partial double blinded RCT	80 hospitalized men ≥ 50 yo	May–June 2020	Mortality rate and hospital stay	Finasteride	Good	No association
**Cadegiani** **et al.** **[96]**	2021	Brazil	RCT	138 SARS-CoV-2 volunteers	June–October 2020	COVID-19-severity outcomes	Dutasteride	Good	Positive association

*: year of publication; **PTS**: patients; **ADT**: androgen deprivation therapy; **AG**: agonist; **ANTAG**: antagonist; **NOS**: not otherwise specified; **GP**: group; **OBS**: observational.

## Data Availability

The data can be shared up on request.

## Supplementary Materials

The following supporting information can be downloaded at: https://www.mdpi.com/article/10.3390/cancers16020298/s1, File S1: Study Quality Assessment Tools.
